# Effect of stress during slaughter on carcass characteristics and meat quality in tropical beef cattle

**DOI:** 10.5713/ajas.19.0804

**Published:** 2020-01-13

**Authors:** Apolo A. Carrasco-García, Violeta T. Pardío-Sedas, Gloria G. León-Banda, Concepción Ahuja-Aguirre, Pedro Paredes-Ramos, Bertha C. Hernández-Cruz, Vicente Vega Murillo

**Affiliations:** 1 Faculty of Veterinary Medicine and Zootecnics, Veracruzana University, C.P. 91710, Veracruz, Veracruz, México

**Keywords:** Bovine, Slaughter, Stress, Cortisol, Meat Quality

## Abstract

**Objective:**

This study aimed to determine the effects of stress during slaughter of beef cattle on physiological parameters, carcass, and meat quality at a Federal Inspection Type slaughterhouse located in the southeast of Mexico.

**Methods:**

A total of 448 carcasses of male Zebu×European steers with an average age of 36 months were included. Carcass assessment of presence of bruises and bruise characteristics was carried out on each half-carcass. Blood variable indicators of stress (packed cell volume, neutrophil to lymphocyte ratio, glucose, cortisol concentration) and meat quality parameters (pH, color, shear force, drip loss) were evaluated.

**Results:**

Of the 448 carcasses evaluated, 81% of the carcasses showed at least one bruise; one bruise was detected in 36.6% and two bruises in 27.0% of animals. Of the 775 bruises found, 69.2% of the bruises were grade 1 in region 3. Of the 448 carcasses studied, 69.6% showed hyperglycemia (6.91 mmol/L); 44.3% and 22.7% showed high (74.7 ng/mL) and extremely high (108.8 ng/mL) cortisol levels, respectively, indicative of inadequate handling of animals during preslaughter and slaughter. Of the carcasses evaluated, 90.4% had a pH ≥5.8 with an average of pH 6.3. In both pH groups, meat samples showed L* values >37.0 (81.6%) and a shear force >54.3 N; meat pH≥5.8 group showed a drip loss of 2.5%. These findings were indicative of dark, firm, and dry (DFD) meat. According to principal component analysis, grades 1 and 2 bruises in region 3 and grade 1 bruises in region 5 were highly associated with cortisol, drip loss, and color parameters b* and h* and were negatively associated with L*, a*, and C*.

**Conclusion:**

The bruises probably caused by stress-inducing situations triggered DFD meat. Appropriate changes in handling routines in operating conditions should be made to minimize stress to animals during the slaughter process to improve animal welfare and meat quality.

## INTRODUCTION

In beef cattle, the hours previous to slaughter are the most stressful of their life and detrimental to the animal’s energy reserves [1]. Stress is mainly experienced during handling and transportation; transport conditions (limited space, long hours of standing), novel/unfamiliar environments (unknown noises and smells), changes in social hierarchies (mixing, mounts, and separation), climatic conditions, and loading, as well as during time spent at the slaughterhouse holding pens and in the passageway to the stunning box [2]. These challenges perturb the animal’s homeostasis, and adaptive response is activated in an attempt to restore balance. Because of these preslaughter-challenges, an animal may experience fear, dehydration and hunger, increased physical activity, fatigue, and physical injury. An autonomic response is typically initiated in reaction to acute stressors. The sympathoadrenal component of autonomic response is mediated by catecholamines (epinephrine and norepinephrine). Activation of the hypothalamic–pituitary–adrenal axis releases glucocorticoids (e.g., cortisol) from the adrenal cortex whose baseline activity is essential for life. Hence, cortisol levels in blood are an important stress indicator in evaluating stress levels [2,3].

The most frequent problems caused by stress in cattle are weight loss, carcass injuries, and alteration of meat quality, particularly due to an increase in pH (>5.8), affecting meat tenderness and color (dark meat) [4]. Meat pH is a result of the amount of glycogen present in the muscles before slaughter, which depends greatly on the factors responsible for physical and psychological stress [5]. Exposure to stressors during slaughter results in ATP reduction, leading to depletion of muscle glycogen concentrations which inversely increases plasma glucose production [6]. Significant depletion of muscle glycogen reserves pre-slaughter has a profound effect on several key meat quality attributes such as pH, tenderness and aging potential, color, and water-holding capacity [7]. A value of pH≥6 at 12 to 48 h postmortem results in dark meat cuts (a defect known as dark, firm, and dry [DFD] meat or dark-cutting beef), which are more susceptible to microbial contamination and have a shorter shelf life and become less acceptable to the consumer as these cuts are dark-red to brown-black and have a dry, firm, sticky consistency [8]. The meat traits that have greater influence on consumer satisfaction are tenderness, juiciness, and flavor of cooked meat, all of which are affected in DFD meat cuts [9].

In Mexico, Federal government slaughterhouses are classified as Federal Inspection Type (FIT) slaughterhouses which must apply federal procedures mandated by Mexican regulations of the Ministry of Agriculture and Rural Development and Ministry of Health to guarantee meat safety. However, the animal welfare and the quality of meat lately have become an important aspect, as an increasing tendency in the presence of DFD meat has been reported. Recently, Loredo-Osti et al [10] showed a frequency of dark-cutting carcasses of 13.45% from bovines slaughtered in a FIT slaughterhouse located at northeastern Mexico. They concluded that risk factors for DFD meat were present in all stages of the slaughter process. In another FIT slaughterhouse located at north of the state of Baja California, Mexico, Pérez Linares et al [8] found a frequency of DFD meat of 13.64% due to management factors. The state of Veracruz is the primary beef producer in Mexico with a commercial beef herd of 4,242,382 heads and 753,615 tons of meat produced annually. Meat is commercialized throughout supermarkets nationwide and exported to the United States, Japan, Vietnam, and Russia. In the state of Veracruz, there are slaughterhouses regulated by municipal authorities and FIT slaughterhouses [11]. Nevertheless, preslaughter handling of livestock has received limited attention. Because there is limited information available on the impact of FIT slaughter practices on animal stress and their influence on carcass and meat quality produced in this important productive region, the aim of this study was to determine the relationship between blood variable indicators of stress and carcass characteristics and meat quality in tropical beef cattle slaughtered at a FIT slaughterhouse in Mexico, to recommend appropriate changes in handling routines that could minimize the biological cost to animals during the preslaughter process and thus improve meat quality.

## MATERIALS AND METHODS

All animal experiments were carried out in accordance with The Mexican Official Norm NOM-033-Z00-2014 Methods of humanitarian slaughter of domestic and wild animals, and associated guidelines [12]. Experiments on live animals were approved by the Bioethics and Animal Welfare Committee of Veterinary Faculty (approval number 151216) confirming compliance with all requirements of Mexico.

### Study location and animals

In order to extrapolate the findings to other FIT slaughterhouses located in the region, the study was conducted at the commercial government-inspected slaughterhouse CIASA FIT 353 located in the central region of the state of Veracruz, Mexico (19°13.4′58″ N, 96°18.38′57″ W, and 39.2 m altitude). This slaughter plant complies with the Official Mexican Norms that regulate the animal care, humanitarian slaughter, and slaughterhouse regulations (NOM-009-ZOO-1994, NOM-024-ZOO-1995, NOM-030-ZOO-1995, NOM-033-ZOO-1995, NOM-194-SSA1-2004) [12]. It has a slaughtering capacity of 7,000 animals per month at a rate of 40 animals per hour. The animals arrived from farms located nearby this FIT slaughterhouse. The average transportation time from these farms to the slaughterhouse was 2.5±0.5 h. The trailers’ conditions complied with the requirements of the Official Mexican Norms for cattle transport (NOM-009-ZOO-1994; NOM-024-ZOO-1995) [12]. The concrete unloading ramps have nonslip floors. They are connected to a lairage area that has ten 128.7-m^2^ pens with asbestos sheet roofing and nonslip concrete floors. In the slaughterhouse, the animals to be evaluated were housed in separate pens and had a rest period from 30 to 90 min. The animals had access to water *ad libitum* but without feed. Electric prods were used for handling practices to herd the animals during their stay in the slaughterhouse. A concrete passageway guided the animals from the lairage area to the stunning area with no head fixation system. Access to the box was through a guillotine door. After being stunned by a pneumatic captive bolt gun (model USSS-1, JARVIS Products Corporation, Middletown, CT, USA), the animals were slaughtered, suspended by a hind leg, and exsanguinated. Afterward, the animals were transferred to the production line to begin the process of removing the head, feet, skin and viscera and quartering the carcass.

### Bruising assessment

**Figure 1:**
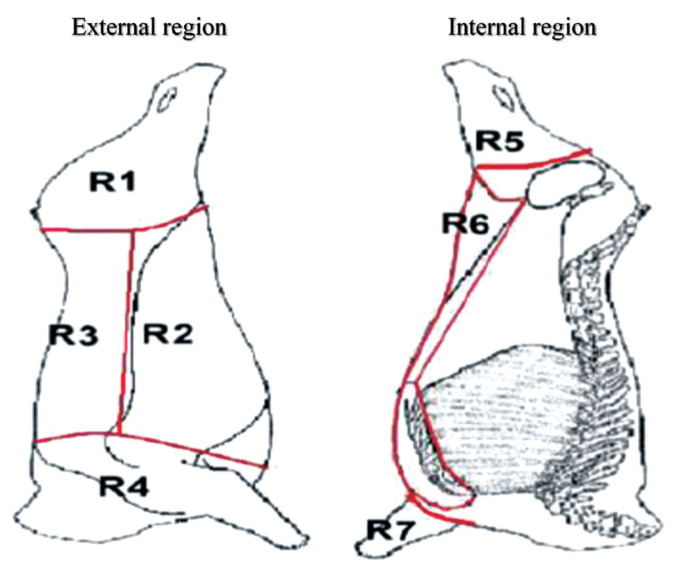
Carcass anatomical regions differentiated for bruising recordings: 1 = lateral side of the pelvic limb, 2 = thorax and abdomen, 3 = thoracic and lumbar vertebrae, 4 = cervical vertebrae and the first five thoracic vertebrae, 5 = medial side of the pelvic limb, 6 = ventral thorax and abdomen, 7 = foreshank/shin [16].

A total of 448 carcasses of male Zebu×European steers with an average age of 36 months and an average live weight of 450±50 kg were included in the study. Only the left half-carcass was evaluated during the study period from January to June 2017. The protocol for the post-mortem evaluation was based on the carcass bruising scoring proposed by Rebagliati et al [13]. Carcass assessment of presence of bruises and bruise characteristics was carried out on each half-carcass divided into four external and three internal regions as shown in Figure 1: region 1 = lateral side of the pelvic limb (meat cuts of high retail value), region 2 = thorax and abdomen (meat cuts of intermediate retail value), region 3 = thoracic and lumbar vertebrae (meat cuts of the highest retail value), region 4 = cervical vertebrae and the first five thoracic vertebrae (meat cuts of low retail value), region 5 = medial side of the pelvic limb (meat cuts of intermediate retail value), region 6 = ventral thorax and abdomen (meat cuts of low retail value), and region 7 = foreshank/shin ( meat cuts of low retail value). Bruise severity was determined considering three levels: grade 1, subcutaneous tissue affected; grade 2, subcutaneous tissue and muscle affected; and grade 3, subcutaneous tissue, muscle, and broken bones.

### Physiological parameters

Four blood variables indicators of stress were evaluated from January to April 2017: packed cell volume (PCV), neutrophil to lymphocyte ratio (NLR), glucose, and cortisol concentrations. Two 10 mL blood samples were collected at slaughter from each animal at the time of exsanguination after stunning; one sample was collected in a 6 mL BD Vacutainer (Becton Dickinson México, México City, México) tube with heparin, and the other sample was collected in a 10 mL tube with 1% NaF. The samples with NaF were centrifuged at 8,000 rpm×5 min, and the serum samples obtained were separated in two aliquots and stored at −20°C until analyzed for cortisol and glucose concentrations.

#### Packed cell volume and neutrophil to lymphocyte ratio

The PCV and NLR analyses were performed using the heparinized samples in an automatic hematology analyzer (COULTER A^c^·T diff; Beckman Coulter, Inc., Brea, CA, USA), following the Coulter principle (counting and sizing).

#### Determination of blood cortisol concentration

Serum cortisol concentration was determined through quantitative radioimmunoassay (RIA), using a commercial kit (RIA Izotop; Institute of Isotopes, Ltd., Budapest, Hungary) [14]. Each sample was determined in duplicate from 10 μL of plasma, with the corresponding controls. The interassay- and intra-assay coefficients of variance were 4.67% and 6.32%, respectively.

#### Determination of blood glucose level

Blood serum glucose concentration was measured using a commercial kit (SPINREACT, glucose LQ, GOD-POD; SPINREACT, S.A.U., Girona, Spain) with a UV-visible spectrophotometer (Spectronic GENESYS 5; Thermo Scientific, Waltham, MA, USA) at 505 nm.

### Meat quality analysis

After 24 h postmortem, a representative sample of one cut of the *longissimus thoracis et lumborum* (LTL) muscle of approximately 15.2 cm long and 2.54 cm thick was collected from the left side of 50 of the 448 carcasses in the meat-packing area (−1°C). Then, cuts were transported to the laboratory in coolers without disrupting the cold temperature to evaluate the following quality parameters.

#### pH determination

At 24 h postmortem, meat pH was determined in the (LTL) muscle of each carcass stored in the cold chamber at −1°C, using an insertion-type portable pH meter (model HI99163; Hanna Instruments, Woonsocket, RI, USA) with a meat puncture electrode, which was inserted into a small incision (4-cm depth) in the (LTL) muscle of the carcass (11th/12th ribs interface). The pH meter was recalibrated after every five samples. Carcasses showing pH values ≥5.8 were classified as DFD and meat was considered normal quality when pH was <5.8, according to the Mexican Ministry of Agriculture regulation [15].

#### Color measurements

Meat color of the raw (LTL) muscle samples was measured at room temperature (25°C) with a Konica Minolta Colorimeter (CR-200 Chroma meter, Osaka, Japan), equipped with pulsed xenon arc lamp with double-beam feedback system, a standard illuminant D_65_, using a 2° position of the standard observer, and 8 mm of reading area. Color measurements were reported in terms of Luminosity (0–100) (L*), index of red (a*), index of yellow (b*), and Chroma (color saturation) (0 to ~85) and hue (color angle) values were determined in the CIELab color space model. Chroma (C*) and hue angle (h*) values were calculated as C* = (a*^2^+ b*^2^)^1/2^ and h* = tan^−^^1^ (b*/a*), respectively [16]. Each measurement for each sample was carried out in triplicate on five preselected locations at the cut surface of each sample.

#### Warner-Bratzler shear force

Three 2.54-cm diameter raw (LTL) muscle sample cores were sheared longitudinally parallel to the muscle fiber, using a hand-held steel core borer. The cores were sheared perpendicular to the muscle fibers using and a TA.XT-plus Texture Analyzer (Stable Micro Systems, Surrey, UK) using a Warner-Bratzler shear device. Assay parameters were as follows: test speed 4.0 mm/seg, down stroke distance 35.00 mm. Shear force values (in Newton [N]) were considered as follows: <22.2 N = tender meat, 22.2 to 35.5 N = moderately tender meat, 35.6 to 53.2 N = tough meat, and >53.2 N = extremely tough meat [17]. Three replicates of WBSF values for each core were averaged for each raw meat sample and used in statistical analyses [15].

#### Drip loss

One 5-g slice (0.5×0.5×3.0 cm) of each raw (LTL) muscle sample was cut longitudinally to the muscle fiber. The slice was placed on a plastic rack in a glass container with absorbent paper at the bottom, covered with plastic film to prevent evaporation and kept at −1°C for 24 h. Then, the slice was removed from the container and reweighed. The amount of drip was expressed as percentage: % drip loss = ([initial weight – final weight]/initial weight) × 100 [18].

### Statistical analyses

Descriptive statistics was used to determine the percentage of bruised carcasses and the total number of bruises in each region of the carcasses by grade. Two distinct groups were formed: pH<5.8 and ≥5.8. These categorical groups were used for the statistical analysis. Differences in blood variables (PCV, NLR, glucose, cortisol) and quality parameters of raw meat (pH, color, shear force, and drip loss) were analyzed using the mixed procedure of SAS [19]. The model to analyze parameters included the fixed effect of categorical pH group (two classes) and the random effect of the animal. The denominator degrees of freedom from the model (DDFM) = Satterth option of the mixed procedure of SAS was used for computing the denominator degrees of freedom for the tests of fixed effects. The DDFM = Satterth option (a general Satterthwaite approximation) implemented here was intended to produce an accurate F approximation. Differences between least squares means for each fixed effect were tested with the probability of difference option of the mixed procedure of SAS. The contribution of stress during slaughter of beef cattle on the carcass and meat quality was analyzed with the principal component analysis (PCA). The PCA was carried out with XLSTAT> 2018 software (Addinsoft; Paris, France) and the level of significance was set at p<0.05.

## RESULTS AND DISCUSSION

### Bruise assessment

As shown in Table 1, the overall number of bruises found in the carcasses was 775; 746 bruises were grade 1, and 29 grade 2; 69.2% of the bruises were grade 1 in region 3. Of the 448 carcasses evaluated, 164/448 (36.6%) presented one bruise, and 121/448 (27.0%) had two bruises; 81% showed at least one bruise. The high frequency is evidence of serious welfare problems in the handling of animals during the preslaughter period in this FIT slaughterhouse. These results agree with those found by Miranda-de la Lama et al [20] who reported that 92% of the carcasses of 8,118 male cattle slaughtered at a commercial plant in the Northwest of Mexico had some type of bruise. Rebagliati et al [13] in Argentina and González [21] in México observed a prevalence of bruises in 37.9% and 32.6% of bovine carcasses, respectively. The percentage of bruises observed in our study could be explained in part by the conditions during transport and the frequent use of electric prods (28.8%) to force animals to move during unloading, indicating that preslaughter management practices were not adequate. Nevertheless, average bruises per carcass were 1.5, lower than the average reported by Rebagliati et al [13] in steers of 2.1.

Table 1 shows the number of bruises by grade found in each region of the carcasses. Of all bruises observed, 96.2% were grade 1, similar to the result obtained by González [21]. Although these bruises do not lead to tissue condemnation, they affect the appearance of the carcass and are considered a good indicator of cattle handling and welfare [13], as these bruises usually result from the equipment and facilities used for handling animals. Of all the bruises observed, 3.8% were grade 2, probably caused by blows to cattle from transportation truck doors or by direct abuse of the animals by their handlers [13]; most of these bruises were causative of tissue condemnation, varying in importance depending on their size and the affected region. No grade 3 bruises were observed. Miranda-de la Lama et al [20] observed 68% of bruises grade 1, 30% grade 2, and 2% grade 3.

Regarding carcass regions in which bruises were present, the location with the highest percentage of bruising (grades 1 and 2) was observed in region 3, from which the most valuable meat cuts are obtained. Bruises in region 3 could be due to the truck doors falling on the back of the cattle when they are passing, or in the passageways leading to stunning area or because of mounting during transportation or during their stay at the slaughterhouse holding pens because the animals are not familiar with them [13]. These results suggest inadequate handling of cattle regarding excessive use or excessive force in the use of herding equipment, such as sticks and electric prods, perhaps due to the lack of trained personnel. Moreover, 93.4% of the bruises were produced in anatomical regions of high retail value, especially the leg, loin, and rib, which correspond to regions 1, 3, and 2, respectively, being region 3 the most affected (71.6% bruises of grade 1 and 2).

### Physiological parameters indicative of stress

Least squares mean and standard errors for PCV, NLR, glucose, and cortisol by categorical pH group are presented in Table 2.

#### Packed cell volume and neutrophil to lymphocyte ratio

The incidence of meat pH<5.8 was 8.9% of the population. No statistical differences (p>0.05) between PCV values of pH groups were observed. Normal values of PCV in cattle are 24% to 46% [5,22]. The PCV increases as a result of stress by transport and handling. Some authors have reported increased PCV in steers after transport to the slaughterhouse. Stress causes a release of catecholamines, which in turn increase blood pressure and causes contraction of the spleen, increasing erythrocyte mobilization into the blood stream [23]. In our study, PCV was within normal values in 98% of the animals; however, as the period from transportation until slaughter was not evaluated, it was not possible to determine if these results were due to the lack of exposure to stress during transportation.

No significant difference (p>0.05) in NLRs between the two groups was detected. The normal value for NLR in cattle is 1.5. NLR values lower than 1.5 result from neutropenia and/or lymphocytosis, while values higher than 1.5 result from neutrophilia and/or lymphopenia, which suggests the presence of stressful, inflammatory or infectious events [5]. In our study, average NLR was 1.7, indicating that the animals may be subjected to stressful situations during slaughter. According to Tadich et al [24], adrenaline is responsible for the neutrophilia and monocytosis that are observed during stressful situations, provoking a decrease in neutrophil mobilization from the blood to the tissues and an increase in the migration of marginal neutrophils found in the blood vessel wall and tissues close to blood circulation.

#### Blood glucose level

The effect of categorical pH group was significant for glucose (p<0.05). Glucose levels in the pH<5.8 group (6.82 mmol/L) were higher (p<0.05) than those in the pH≥5.8 group (5.64 mmol/L). Considering that normal values of glucose in cattle are 3.3 to 4.6 mmol/L [22], 69.6% of the 448 carcasses evaluated showed hyperglycemia, 21% had normal glucose values, and 9.4% showed hypoglycemia. Short fasting periods produce hypoglycemia; this triggers catecholamine release, which promotes glycolysis and gluconeogenesis, resulting in hyperglycemia [25]. Tadich et al [24] mentioned that fasting during transportation resulted in higher blood glucose and cortisol levels, PCV, and leukocytes than fasting during confinement.

#### Blood cortisol concentration

No statistical differences (p> 0.05) between cortisol values of pH<5.8 group (75.33 ng/mL) than those observed in the pH≥5.8 group (63.89 ng/mL) were observed. Similar results were reported by Poleti et al [26] who found cortisol levels of 61.6 and 70.2 ng/mL between high (pH≥6.0) and normal (pH<5.8) ultimate pH groups, respectively, in bovine *Longissimus thoracis* muscle. In our study, the percentages of animals with normal (25 to 45 ng/mL), high (>45 – 90 ng/mL), or extremely high (>90 ng/mL) [3] cortisol levels were 23.2% (36.1 ng/mL), 44.3% (63.0 ng/mL), and 22.7% (108.8 ng/mL), respectively. This suggested that the animals faced stressful conditions. The percentages (44.3% and 22.7%) of the animals that showed high and extremely high levels of cortisol can be attributed to the inadequate handling of animals during preslaughter and slaughter. Moreover, animals had a rest period from 30 to 90 min, but 90.4% of the animals showed a pH≥5.8. Moreover, some animals were not properly stunned, because palpebral reflex and vocalizations were observed after stunning, and these animals were not stunned again. As this stressful situation was of short duration and happened immediately prior to death, it might not be reflected in the cortisol levels, which increase approximately 15 min after exposure to the stressful stimulus [25]. In contrast, when animals are exposed to stressful stimuli since the beginning of the fattening period, they manifest chronic stress, and thus, cortisol levels may be normal due to adaptation of the animal to the stressor. However, this effect may be reflected in the glucose levels, as glucose takes longer to increase, but also to decrease to normal levels after adaptation [24].

### Quality parameters of raw meat

Least squares mean and standard errors for quality parameters pH, L*, hue*, chroma*, shear force and drip loss by categorical pH group analyzed 24 h postmortem are presented in Table 3.

#### pH determination

Of the 50 carcasses evaluated, 10 (20.0%) had pH<5.8 and 40 (80.0%) a pH≥5.8. The effect of categorical pH group was significant for pH (p<0.05), as the mean raw meat pH values (6.3) of the pH≥5.8 group were higher (p<0.05) than those of the pH<5.8 group (5.6). pH measurement is one of the most important quality parameters of meat quality as it is related to depletion of muscle glycogen reserves. After animal death by exsanguination, muscles become anoxic, triggering anaerobic glycolysis. High levels of stress hormones before or during slaughter decrease muscular glycogen reserves, as glycogen is hydrolyzed to lactic acid. Therefore, meat pH decreases from 7.0 to 5.5 are essential for reduction of bacterial growth [3,23]. Cortisol levels may play a role in correct acidification of meat. Therefore, to avoid adverse effects on the carcass, such as decreased shelf life, color alterations and decreased tenderness due to pH values >5.5, management of animal welfare during growth, transport, and slaughter are pivotal. According to Mexican regulation pH values ≥5.8 are related to DFD meat. This condition would result in poor-quality meat cuts and shorter shelf life [15].

#### Color measurements

According to Table 3, hue* and chroma* variables were affected by the categorical pH group (p< 0.05); samples of pH<5.8 meat had higher hue* and chroma* mean values than those in pH≥5.8 meat. The mean value of complementary colors red green (a*) coordinate of 13.55 units of pH<5.8 meat was higher (p<0.05) than 11.05 units of pH ≥5.8 meat. Similarly, the mean calculated for complementary colors yellow green coordinate (b*) of pH<5.8 meat was 9.3 units, which was higher (p<0.05) than the 5.5 units of pH≥5.8 meat. According to Abril et al [27], pH≥6.1 meat from male cattle had lower L*, a*, b*, C*, and h* values than pH<6.1 meat at the most measuring times; this is characteristic of the darker and redder color of DFD meats. Poleti et al [26] observed that meat with high pH≥6.0 had lower L*, a*, and b* values within all postmortem times, indicating that muscle with high pH was darker, less red, and less yellow. During rigor onset, the increase in the overlapping myofilaments and shortening of the sarcomere is associated with higher reflectance. As pH falls, there is an increase in birefringence, more light scattering, reduced transmittance and a proposed increase in lightness value. Thus, in DFD, the muscles absorb light, making the meat appear darker. A darker meat of high pH could be attributed to a lower amount of free water at its surface and a lower oxygenation of myoglobin [28]. When animals are exposed to chronic or long-term stress before slaughtering, DFD meats can occur. The study of Loredo-Osti et al [10] revealed that the probability of risk for DFD meat increased 5.3% for each our spent in the waiting pens prior to slaughter, and after slaughter, a ΔpH value of 0.5 units represented a probability of observing dark-cutting carcasses equal to 79.7%. As previously mentioned, the animals in our study showed high and extremely high levels of cortisol and had a rest period from 30 to 90 min. According to the model, a significant (p< 0.05) increase of pH in meat was associated with a decrease in b* (*r* = −0.546) and C* (*r* = −0.480) values. In our study, 81.6% of the animals had L* values within a range of 39.5 to 40.3. According to Muchenje et al [29], L* values of 37.0 to 40.4 can be considered DFD in beef. It has been suggested that L*, which denotes lightness, could be used as an indicator as it has shown to be the most important parameter related to DFD. Thus, meat that is dark may cause substantial economic loss to the meat industry worldwide. Color is the most important sensory attribute of meat that influences consumer purchasing decisions because consumers associate the bright cherry red color with freshness and quality, and any deviation from this is perceived as degradation in quality. Holman et al [30] found that an L* value between 14.5 and 44.0, a* between 3.3 and 14.8 (which increases as L* increases), and an optimal b* value of 19 in beef are critical to consumer acceptability. Thus, consumers would probably consider the beef samples of our study acceptable according to L* and a* values, but not for b* values.

#### Warner-Bratzler shear force

As shown in Table 3, no significant difference (p>0.05) between pH groups was observed. The precise shear force value at which a carcass is deemed DFD meat at ultimate pH is >54.3 N, which corresponds to a tough meat [31]. Of the samples analyzed, 92% showed tenderness values >54.3 N. Thus, tenderness found in both pH groups corresponded to DFD meat. According to the model, the interaction of shear force and L* values of the pH≥5.8 group was correlated (*r* = 0.928) (p>0.05). There is an increased incidence of tough meat and decreased lightness between pH values of 5.8 and 6.3 [32]. In the pH range of 5.8 to 6.2 less tender meat is generated; this has been attributed to low titin and nebulin degradation rates. According to Purchas [33], DFD beef with intermediate pH values (5.8 to 6.2) had the highest shear force values due to decreased sarcomere length. Reduced sarcomere length is recognized as an important cause of increased toughness in meat, and it appears that sarcomeres increase in length as pH decreases below 6.2. Furthermore, tenderness is affected by temperament ranking as increasing excitability is associated with higher (p<0.05) serum cortisol concentrations The *B. indicus* genotype is commonly responsible for an important variation in tenderness, because the meat from *B. indicus* breeds is tougher than meat from *B. taurus* breeds [34]. Animals that had very excitable temperaments, such as *B. indicus*, produced steaks with shear force values >104.9311 N when compared with calm temperaments with shear force values <50.9946 N. The threshold value for acceptability in food service establishments is 38.2459 N as beef tenderness is a primary factor determining customer satisfaction [35]. It is important to note that the animals in our study were *B. indicus* × *B. taurus* and Warner-Bratzler shear force values for both pH groups were higher than these thresholds. Furthermore, these shear force values were higher than those recommended by Mexican regulations [15]: <31.3813 N = tender meat, 31.3813 to 38.2459 N = moderately tender meat, and >39.2266 N = tough meat, and thus meat would be considered tough or DFD.

#### Drip loss

As shown in Table 3, no significant difference (p>0.05) between pH groups was observed. A mean moisture loss from dripping of 2.4% is indicative of DFD meat [15]. According to the model, drip loss values of the pH≥5.8 group were related (*r* = 0.990) to L* values (p>0.05), possibly associated to shortening of the sarcomere and associated with higher reflectance. Ultimate meat pH and rate of pH decline are influenced by biochemical events premortem- and postmortem, which act on structural components in muscle cells [36]. As 85% of the water in a muscle cell is held in the myofibrils, drip loss is influenced by interfilament spacing and the extent of lateral and transverse shrinkage of myofibrils at rigor, postmortem cytoskeletal protein degradation, the development of drip channels and extracellular space, and the permeability of the cell membrane to water [36]. In our study, the carcasses were stored at temperature below 10°C when their pH was still >6.0; cooling could have caused muscle fiber to shorten, although it may also have been caused by previous electrostimulation during the slaughter process. Hargreaves et al [37] mentioned that the cooling rate of the carcass influences pH and, therefore, the occurrence of DFD meat.

**Figure 2:**
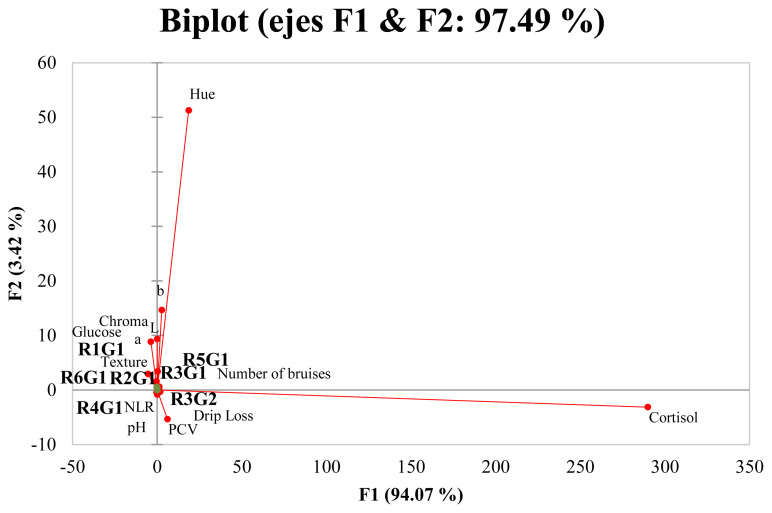
Principal component analysis projections of scores and loadings for the first two principal components for the analysis of bruises and physiological and meat quality parameters. Variables with vectors projected in the same plane may be considered to be positively correlated.

Principal component analysis indicated that two principal factors described 97.49% of the variation between variables (Figure 2). The first factor accounted for 94.07% of the total variation and primarily described the effect of high cortisol levels on color parameters b* and h* values, physiological parameter PCV, quality parameter drip loss, number of bruises (animals with two to four bruises), and grades 1 and 2 bruises in region 3 and grade 1 bruises in region 5. These types of bruises, however, had a negative association with color parameters L*, a*, and C*, with values indicative of DFD meat. As these numbers and bruise types were highly associated with cortisol, it can be inferred that these bruises were probably caused by stress-inducing situations during preslaughter and slaughter. Accordingly, if the stress response is strong enough, meat quality will be affected. In contrast, factor F2 represented 3.42% of the remaining variance related to the increase in pH, glucose, texture, and changes in color parameters L*, a*, and C* values. It is important to point out that F2 comprised grade 1 bruises in regions 1, 2, 4, and 6 and the number of bruises (animals with zero to two bruises), indicating that although these types of bruises were low in number, they produced stress as well. The current Federal Norm (NOM-033-SAG/ZOO-2014) and the World Organization for Animal Health (Office International des Epizooties) [38] state that cattle must be handled according to animal welfare guidelines before slaughter and animals should be slaughtered as soon as possible after arrival to the slaughterhouse. In the present study, a 30- to 90-min lairage period, palpebral reflex and vocalizations after stunning, and excessive use of the electric prods were observed, indicating that the personnel responsible for the cattle during the preslaughter period is not trained and certified on the humane handling of cattle.

In conclusion, under the conditions of this study, carcasses with pH≥5.8, low L* values, high shear resistance and drip loss values, were indicative of DFD meat. High plasma cortisol levels at exsanguination during slaughter were highly associated with the numbers and bruise types, color parameters and drip loss values. These results suggest the exposure of cattle to stressful situations before and during the slaughtering process, a poor infrastructure design, and not well trained staff. Therefore, handling routines and/or training of the operators in commercial operating conditions should be achieved to minimize the biological cost to animals during the slaughter process and to improve the meat quality.

## Figures and Tables

**Table 1:** Number of bruises found in the carcasses evaluated and total number of bruises in each region of the carcasses by grade

Number of bruises	Number of carcasses (%)	Region	Grade-1 n (%)	Grade-2 n (%)	Total n (%)
0	85 (19.0)	1	75 (9.7)	3 (0.4)	78 (10.1)
1	164 (36.6)	2	85 (11.0)	6 (0.8)	91 (11.7)
2	121 (27.0)	3	536 (69.2)	19 (2.5)	555 (71.6)
3	46 (10.3)	4	35 (4.5)	1 (0.1)	36 (4.6)
4	20 (4.4)	5	11 (1.4)	0 (0)	11 (1.4)
5	3 (0.7)	6	2 (0.2)	0 (0)	2 (0.3)
6	2 (0.5)	7	2 (0.2)	0 (0)	2 (0.3)
7	5 (1.1)				
8	1 (0.2)				
9	1 (0.2)				
Total	448		746 (96.2)	29 (3.8)	775 (100)

**Table 2:** Values of blood parameters indicative of stress in cattle during pre-slaughter by pH group

Parameter	pH<5.8	pH≥5.8
PCV (%)	36.71±0.73^a^	35.28±0.23^a^
NLR	0.76±0.06^a^	0.80±0.02^a^
Glucose (mmol/L)	6.82±0.34^a^	5.64±0.10^b^
Cortisol (ng/mL)	75.33±6.06^a^	63.89±1.93^a^

PCV, packed cell volume; NLR, neutrophil/lymphocyte ratio.

a,b Least-squares means within a row with different letters differ (p<0.05) between pH values.

**Table 3:** Quality parameters for raw meat of beef cattle by pH group

Parameter	pH<5.8	pH≥5.8
pH	5.6±0.10^b^	6.3±0.10^a^
L^*^	40.3±0.02^a^	39.5±0.03^a^
hue^*^	35.0±0.08^a^	26.2±0.06^b^
Chroma^*^	16.3±0.01^a^	12.4±0.04^b^
Shear force (Newton)	69.8±0.09^a^	67.5±0.10^a^
Drip loss (%)	2.5±0.07^a^	2.3±0.06^a^

a,b Least-squares means within a row with different letters differ (p<0.05) between pH values.
